# Explicit and Implicit Positive Alcohol Expectancies in Problem and Non-Problem Drinkers: Differences Across Age Groups from Young Adolescence to Adulthood

**DOI:** 10.3389/fpsyg.2015.01773

**Published:** 2015-11-17

**Authors:** Aurélie Vilenne, Etienne Quertemont

**Affiliations:** Département Psychologie, Cognition et Comportement, Université de LiègeLiège, Belgium

**Keywords:** age differences, alcohol expectancies, implicit associations, adolescence, stimulation, sedation

## Abstract

**Aims:** Recent studies with animal models showed that the stimulant and sedative effects of alcohol change during the adolescent period. In humans, the stimulant effects of ethanol are most often indirectly recorded through the measurement of explicit and implicit alcohol effect expectancies. However, it is unknown how such implicit and explicit expectancies evolve with age in humans during adolescence.

**Methods:** Adolescent (13–16 year old), young adult (17–18 year old), and adult (35–55 year old) participants were recruited. On the basis of their score on the Alcohol Use Disorder Identification Test (AUDIT), they were classified as non-problem (AUDIT ≤ 7) or problem (AUDIT ≥ 11) drinkers. The participants completed the Alcohol Expectancy Questionnaire (AEQ) and performed two unipolar Implicit Association Test (IAT) to assess implicit associations between alcohol and the concepts of “stimulation” and “sedation”.

**Results:** Problem drinkers from the three age groups reported significantly higher positive alcohol expectancies than non-problem drinkers on all AEQ subscales. Positive alcohol explicit expectancies also gradually decreased with age, with adolescent problem drinkers reporting especially high positive expectancies. This effect was statistically significant for all positive expectancies, with the exception of relaxation expectancies that were only close to statistical significance. In contrast, stimulation and sedation alcohol implicit associations were not significantly different between problem and non-problem drinkers and did not change with age.

**Conclusion:** These results indicate that explicit positive alcohol effect expectancies predict current alcohol consumption levels, especially in adolescents. Positive alcohol expectancies also gradually decrease with age in the three cross-sectional groups of adolescents, young adults, and adults. This effect might be related to changes in the physiological response to alcohol.

## Introduction

Recent studies with animal models indicated that the stimulant and sedative effects of alcohol are age-dependent. During development, the stimulant effects of alcohol gradually decrease from weaning to adulthood, whereas the sedative effects of alcohol increase with age during the same developmental period (Silveri and Spear, 1998; Stevenson et al., 2008; Quoilin et al., 2010). In humans, this would suggest that young adolescents are more likely to feel the stimulant effects of alcohol, whereas they are less likely to experience its sedative effects, making alcohol even more enjoyable than in adults. Such effects might contribute to promote heavy alcohol drinking in young people, leading to alcohol problems later in life. Unfortunately, very few human studies attempted to directly measure the stimulant and sedative effects of ethanol (Davidson et al., 2002; van den Wildenberg et al., 2006; Addicott et al., 2007) and to our knowledge none of those studies compared participants of various ages including young adolescents. In human studies, it is much more common to measure alcohol effect expectancies through questionnaires than directly recording the behavioral effects of alcohol after consumption. Typically, alcohol drinkers are asked to fill in a questionnaire about how they think alcohol affects the behavior. In these questionnaires, some of the questions pertain to the stimulant and depressant effects of alcohol. One example is the Alcohol Expectancy Questionnaire (AEQ; Brown et al., 1987). For example, in this questionnaire, participants are asked to state how much they agree with the following item: “alcohol increases arousal; it makes people feel stronger and more powerful”. Of course, one of the problems with such expectancy questionnaires is that general expectancies about alcohol consumption may differ from the actual behavioral responses after alcohol consumption. However, probably due to their ease of use, many human studies use alcohol effect expectancies to indirectly infer the stimulant and depressant effects of alcohol.

Many previous studies were interested in explicit positive alcohol effect expectancies as explanatory factors for alcohol-related problems both in adults (Jones et al., 2001) and in adolescents (Baer, 2002). In particular, alcohol explicit expectancies of arousal measured with questionnaires such as the AEQ were shown to predict current alcohol consumption (Kreusch et al., 2013). These expectancies appear to contribute both to the initiation and maintenance of drinking behaviors. Indeed, positive alcohol expectancies predict current alcohol consumption and to a smaller extent future alcohol consumption and alcohol-related problems. They also show consistent associations with problem drinking behaviors in young adults (Cameron et al., 2003; Ham and Hope, 2003). According to the alcohol expectancy theory, individuals who expect positive outcomes to occur as a result of alcohol use are more likely to drink than those who do not endorse such beliefs. As a consequence, positive expectancies, such as arousal expectancies, assessed with the AEQ (Brown et al., 1987) are associated with a higher prevalence of alcohol-related problems, whereas negative expectancies tend to limit alcohol consumption, leading to the opposite effect (for review, Jones et al., 2001). Indeed, heavier drinkers in general report more positive expectancies than light drinkers (Southwick et al., 1981). In a recent study, it was shown that heavy drinkers endorse more positive and arousing alcohol effect expectancies than light drinkers, although heavy and light drinkers do not differ in their endorsement of negative alcohol expectancies (Field and Wiers, 2012). Although all alcohol consumers show positive alcohol expectancies, high arousal expectancies were reported especially in heavy drinkers (Wiers et al., 2002; Field and Wiers, 2012). Therefore, explicit expectancies of alcohol stimulation seem to differentiate low drinkers from high drinkers (Goldman et al., 1999).

One caveat with explicit alcohol effect expectancies is that their measurement relies on self-reports and therefore are associated with many potential biases such as self-representation, limits in introspection aptitudes, and social desirability (Edwards, 1958; Arnold and Feldman, 1981; Fisher, 1993; Greenwald et al., 2002). For these reasons, more indirect assessment of expectancies were developed using a variety of tasks, most of them relying on the measurement of reaction times. For example, the Implicit Association Test (IAT) has been modified to assess the strength of alcohol implicit cognitions. The IAT is a categorization task used to assess automatic associations between concepts. Recent studies used the IAT to measure implicit alcohol associations on two dimensions: valence (positive–negative) and arousal (arousal–sedation). Whereas both light and heavy drinkers showed negative implicit associations with alcohol, the arousal dimension discriminated heavy drinkers from light drinkers. Strong associations between arousal/stimulation and alcohol were found in heavy drinkers but not in light drinkers (Wiers et al., 2002; Field and Wiers, 2012). However, another recent study, while also finding an implicit association between alcohol and arousal with the IAT, failed to show a correlation between such an implicit association and the current levels of alcohol consumption in young adults (Kreusch et al., 2013). As alcohol stimulant effects are expected to decrease with age, the age of participants might explain such a failure to find a correlation between alcohol consumption and IAT implicit alcohol associations.

Alcohol use behaviors typically change over time, especially during the transition period from adolescence to adulthood, with peak years for the initiation of drinking near 13–14 years old (Faden, 2006). Rates of alcohol use and intoxication increase during adolescence and continue to rise in the years after high school (O’Malley et al., 1998; Brown et al., 2008). Alcohol use during adolescence, particularly heavy drinking, has been associated with alcohol misuse and dependence later in life (Grant and Dawson, 1997; Cable and Sacker, 2008). These changes in alcohol drinking behaviors might be associated with changes in alcohol effect expectancies, which might in turn affect subsequent alcohol consumption. Expectations about the effects of alcohol were reported in children even before they begin to drink alcohol (Miller et al., 1990; Field and Wiers, 2012), suggesting that such expectations might influence the initiation of alcohol consumption. In young adolescents, both explicit and implicit alcohol-related cognitions were reported to influence drinking behaviors (Thush and Wiers, 2007). Furthermore, positive alcohol expectancies predicted all types of adolescent alcohol use in young men and women (Cable and Sacker, 2008). Positive expectancies were related more strongly than negative expectancies to drinking in younger age groups, while in the older groups, positive and negative expectancies were both influential (Leigh and Stacy, 2004). However, the ability of alcohol expectancies to predict subsequent drinking seems to diminish with age. Although they are robust predictors of subsequent alcohol drinking in young subjects, their predictive value is reduced in older age groups. Together, these results suggest that the role of alcohol expectancies in alcohol drinking and abuse may fluctuate with age.

The aim of the present cross-sectional study was to assess how implicit and explicit alcohol expectancies differ in various age groups from adolescence to adulthood, with a special focus on arousal expectancies that were previously shown to discriminate heavy from light drinkers (Goldman et al., 1999; Wiers et al., 2002). Participants were also classified as problem and non-problem drinkers in order to see how alcohol expectancies predict current levels of alcohol consumption in participants and whether this relationship changes across age groups. Alcohol explicit expectancies were measured with the AEQ questionnaire (Vautier and Moncany, 2008), whereas alcohol implicit associations were tested with the IAT (Greenwald et al., 2003). In order to avoid a multiplication of IAT tests, the assessment of alcohol implicit associations were limited to the arousal–sedation dimension that was previously shown to discriminate light and heavy drinkers.

## Materials and Methods

### Participants

A total of 105 participants (53 women and 52 men) from three age groups were recruited for the study among university students and their relatives at the University of Liège, Belgium. Adolescent, young adult and adult participants were, respectively, 13–16, 17–18, and 35–55 years old. Adolescents were selected at the age of 13–16 because this age range includes the peak years for the initiation of alcohol drinking (Faden, 2006). The participants were first invited to fill in the Alcohol Use Disorders Identification Test (AUDIT; Saunders et al., 1993). On the basis of their AUDIT score, they were classified as non-problem drinkers (AUDIT score of maximum 7) or problem drinkers (AUDIT score of 11 or above). Additionally, in order to exclude total abstainers or adolescents who had never drunk alcohol, only participants who consumed at least one alcohol drink per week were included in the non-problem drinker group. An appointment for the experiment was made with participants meeting these inclusion criteria. The Symptom Checklist-90 (SCL-90; Derogatis, 1977), assessing psychiatric symptoms, was also used for exclusion of participants with scores of 70 or higher on any SCL-90 subscale. Regular use of other psychoactive substances (except nicotine) was also used as an exclusion criterion. Demographic data are shown in **Table 1**.

**Table 1:** Mean age and Alcohol Use Disorder Identification Test (AUDIT) score and number of binges on the last 2 weeks (SD) for each condition.

	Age		
Consumption level	Adolescents	Young adults	Adults
Low drinkers	*N* = 19 (7 women) Age: 14.89 (0.994) AUDIT: 3.37 (1.862) Binge: 0.26 (0.45)	*N* = 18 (14 women) Age: 17.33 (0.485) AUDIT: 3.33 (2.058) Binge: 0.56 (0.7)	*N* = 17 (10 women) Age: 44.64 (4.859) AUDIT: 4.47 (2.095) Binge: 0.76 (1.2)
High drinkers	*N* = 11 (6 women) Age: 15.54 (0.687) AUDIT: 15.72 (4.36) Binge: 1.27 (1.1)	*N* = 24 (10 women) Age: 17.5 (0.51) AUDIT: 13.13 (2.332) Binge: 1.67 (1.2)	*N* = 16 (6 women) Age: 46.94 (4.626) AUDIT: 13.63 (3.096) Binge: 1.5 (1.8)

### Design and Procedure

Participants were tested one by one in a small and quiet room. They first signed a consent form prior to their participation and filled in some of the questionnaires, i.e., demographic data, and alcohol consumption questionnaires. Then, they performed the IAT. At the end of the task, they filled in the rest of the questionnaires, i.e., the AEQ and the SCL-90. The procedure was approved by the Ethical Committee of the Psychology Faculty from the University of Liège.

### Recent Alcohol Consumption

Recent alcohol consumption was assessed with a self-reported measure based on the timeline follow-back method (Sobell and Sobell, 1992). Participants reported how many standard alcohol drinks (=10 g) they had consumed during the previous week by indicating how many alcoholic drinks they had drunk on each day of the week. Furthermore, the number of days they drank more than six drinks of alcohol (=60 g) on a single occasion during the past 2 weeks was recorded (Wiers et al., 1997). This self-reported assessment of recent alcohol use is a complement to the measurement of habitual alcohol consumption provided by the AUDIT.

### Implicit Association Test (IAT)

The IAT (Greenwald et al., 2003) was used to assess implicit associations between alcohol and two concepts: stimulation and sedation (Kreusch et al., 2013). Participants performed two unipolar variants of the test during which they had to classify stimuli in two categories (i.e., alcohol and soft drinks) and two attribute categories (i.e., stimulation/sedation and neutral), using a left and a right response key. The items used for each category are listed in **Table 2**. An IAT effect is observed when the participants are faster to respond when highly associated categories are assigned the same response key. Each IAT session consisted of seven blocks. In the first and second blocks (24 trials), participants, respectively, practiced classifying target stimuli as “alcohol drink” or “soft drink” and attributed stimuli into the categories “neutral” or “stimulant” (“sedation” for the sedation IAT). In the third (practice, 12 trials) and fourth (test, 48 trials) blocks, participants had to classify stimuli belonging to one target category and one attribute category (e.g., “alcohol drink” and “neutral”) with one response key and stimuli belonging to the other target category and attribute category (e.g., “soft drink” and “stimulant”) with the other response key. In the fifth block (24 trials), participants practiced the reversed response assignment of the target categories. In the sixth (practice, 12 trials) and seventh (test, 48 trials) blocks, participants had to classify stimuli belonging to one target category and one attribute category in the reverse order (e.g., “soft drink” and “neutral”) with one response key and stimuli belonging to the other target category and attribute category (e.g., “alcohol drink” and “stimulant”) with the other response key. The assignment of the categories to the left and the right response keys and the order of the combined sorting conditions were counterbalanced across participants. Stimuli were shown in the middle of the computer screen, and the labels of the categories were presented in the upper corners, consistent with the response assignment of the categories. The stimulus word remained on the screen until a response was generated. Categorization errors were signaled with the word “*error*” appearing in front of the stimulus item. All IATs were programmed in E-Prime 2.0 (Psychology Software Tools, Inc., Sharpsburg, PA, USA). To assess the IAT effect, we used the *D*-score as recommended by Lane et al. (2007). Internal consistencies were tested by calculating the correlation between two *D*-scores (from the third training and the fourth test blocks). The internal consistency was 0.79 for the alcohol-stimulation IAT and 0.67 for the alcohol-sedation IAT.

**Table 2:** Items (in French) used for each Implicit Association Test (IAT) target and attribute category.

Soft drink	Alcohol drink	Stimulation	Sedation	Neutral
Jus d’orange Cacao Coca Limonade Soda Orangeade	Tequila Whisky Vodka Vin Bière Rhum	Vigoureux Excité Agité Energique Actif Eveillé	Passif Tranquille Détendu Apaisé Relaxé Endormi	Normal Indéfini Général Ordinaire Indifférent Moyen

### Alcohol Use Disorders Identification Test (AUDIT)

The French version of the AUDIT questionnaire (Gache et al., 2005) includes ten multiple-choice items measuring alcohol consumption (questions one to three), dependence (questions four to six) and alcohol-related problems (questions seven to ten; Saunders et al., 1993). Questions one to eight are scored from zero to four and questions nine and ten are scored zero, two, or four. The maximum score on the AUDIT is 40. In the present study, a cut-off score of 11 was set for the recruitment of problem drinkers (Fleming et al., 1991) and a cut-off score of maximum 7 for non-problem drinkers, who are also usually qualified as low risk drinkers (Saunders et al., 1993).

### Alcohol Expectancy Questionnaire (AEQ)

We used a validated French version of the AEQ (Vautier and Moncany, 2008), which included 55 self-reported items measuring positive alcohol expectancies. The AEQ includes six subscales: global positive changes, sexual enhancement, social and physical pleasure, arousal/power, social assertiveness, and relaxation. The score on each subscale was calculated by summing the scores obtained on each item (from 0 “*I do not agree*” to 10 “*I totally agree*”). An example of item of the AEQ looks like: “*Alcohol makes me worry less*”.

### Symptom Checklist-90 – Revised (SCL-90-R)

The French version of the SCL-90-R (Fortin and Coutu-Wakulczyk, 1985) is a 90-item self-administered psychopathological assessment questionnaire. The SCL-90-R includes nine subscales: somatization, obsessive–compulsive, interpersonal sensitivity, depression, anxiety, hostility, phobic anxiety, paranoid ideation, and psychoticism. It includes 90 items with five alternatives for each item ranging from 0 (none) to 4 (very much). The SCL-90-R scores are converted to standard *T*-scores.

### Data Analysis

To control for the homogeneity of psychopathological symptoms in the six groups, a two-way analysis of variance (ANOVA; Age group × Consumption group) was computed on the SCL-90 subscale scores. The gender distribution of participants in the six experimental groups was checked using Pearson chi-square tests. The AEQ scores were tested using a multivariate analysis of variance (MANOVA) with Age and Consumption groups (problem vs. non-problem drinkers) as between-subject independent variables and the AEQ subscales as dependent variables. A statistically significant MANOVA was followed by independent two-way (Age × Consumption group) ANOVAs for each AEQ subscale separately. In order to test whether there is an IAT effect for alcohol stimulation and sedation in the whole sample, IAT results were first analyzed with student *t*-tests. The IAT scores on both subscales were then tested using two-way (Age × Consumption group) ANOVAs. Simple eta-squared (η^2^) were reported as effect sizes in the ANOVAs. All statistical analyses were performed using the software package Statistica 10 (StatSoft, Inc., Maisons-Alfort, France). Statistical significance was set at *p* < 0.05.

## Results

### Group Homogeneity

The two-way ANOVA computed on the SCL-90 scores showed no significant main effect of the consumption group (problem vs. non-problem drinkers) and no significant interaction Age × Group for any of the SCL-90 subscales. These results confirm that the problem and non-problem drinkers did not differ on mean levels of psychopathology symptoms in the present study. In addition, there was no significant main effect of age on any of the SCL-90 subscale scores, except for hostility and phobia. Indeed, adolescents globally showed higher mean scores than young adults and adults on these two SCL-90 subscales. As there was an effect of age on hostility and phobia subscales, the two-way ANOVA computed on AEQ and IAT scores (see below) were followed by analyses of covariance in which hostility and phobia scores were included as covariates. These covariance analyses led to identical effects and conclusions than the ANOVAs, confirming that hostility and phobia differences do not explain the effects of age in these analyses. Additionally, experimental groups did not significantly differ regarding gender, χ^2^(5) = 9.15, *p* = 0.10.

### Effects of Age and Alcohol Consumption on AEQ Scores

**FIGURE 1:**
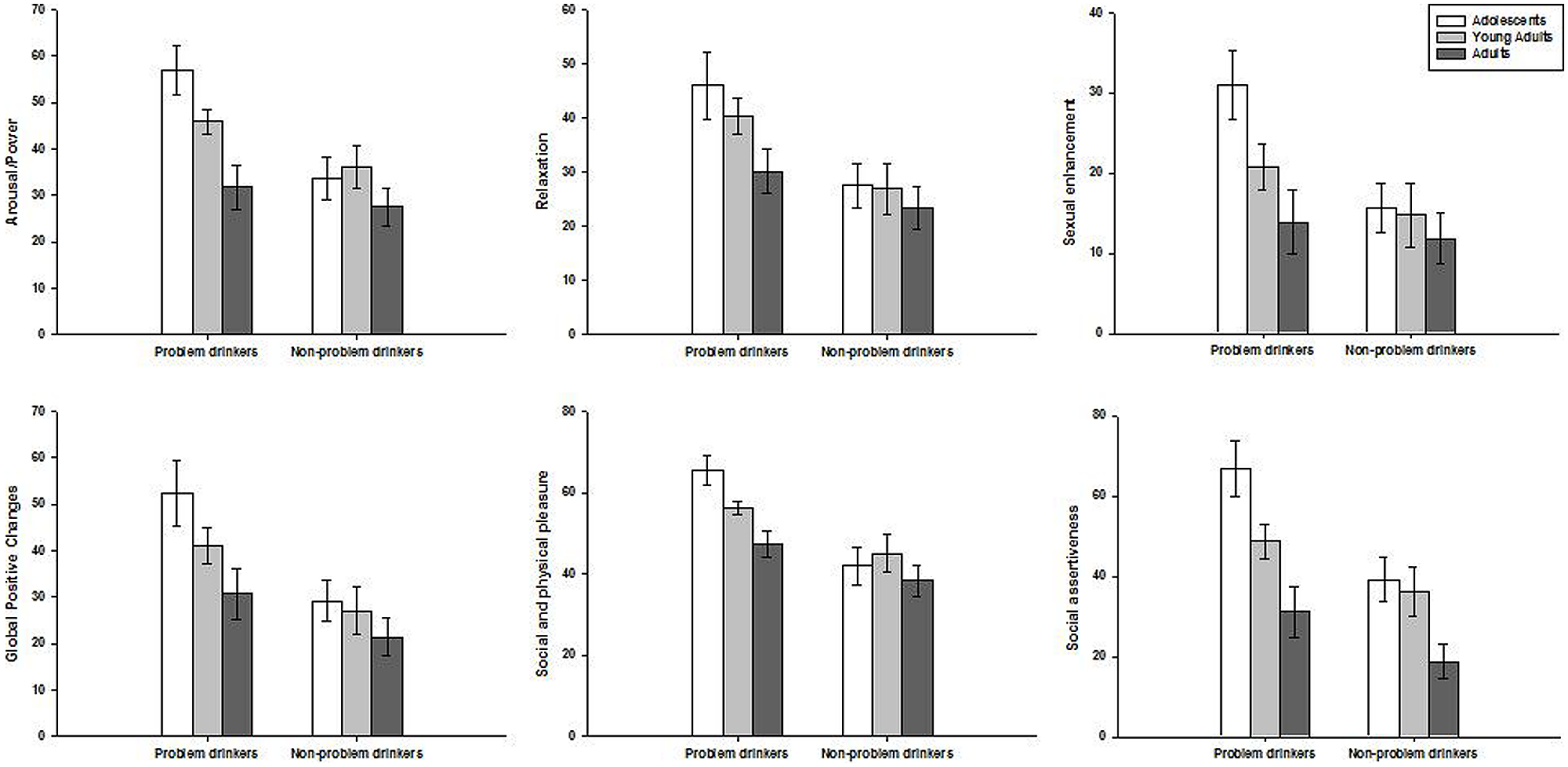
**Mean scores (±SEM) on the Alcohol Expectancy Questionnaire (AEQ) subscales according to age (adolescents, young adults, and adults) and alcohol consumption groups (problem and non-problem drinkers)**.

The MANOVA computed on AEQ scores showed a significant main effect of the consumption group [*F*(6,94) = 3.69; *p* = 0.0025] and a significant main effect of age [*F*(12,188) = 2.48; *p* = 0.005], whereas the interaction was not significant [*F*(12,188) = 0.74; *p* = 0.71]. Two-way ANOVAs were then computed on AEQ subscale scores. There were significant main effects of the consumption group and age for most of the subscales except for the relaxation scale, whereas none of the interactions were statistically significant. The details of these analyzes are given in **Table 3**. As shown on **Figure 1**, problem drinkers reported higher mean positive expectancies about the effects of alcohol than non-problem drinkers on all AEQ subscales. Additionally, mean positive expectancies gradually decreased with age on all AEQ subscales, although the effect of age failed to reach statistical significance on the relaxation subscale.

**Table 3:** Results of the two-way ANOVA computed on the Alcohol Expectancy Questionnaire (AEQ) subscale scores.

AEQ subscale	Age	Alcohol group	Interaction
Positive changes	*F*(2,99) = 4.08; *p* = 0.020, η^2^= 0.07	*F*(1,99) = 14.61; *p* = 0.00023, η^2^= 0.12	*F*(2,99) = 0.92; *p* = 0.40, η^2^= 0.02
Sexual enhancement	*F*(2,99) = 3.81; *p* = 0.026, η^2^= 0.07	*F*(1,99) = 6.89; *p* = 0.010, η^2^= 0.06	*F*(2,99) = 1.60; *p* = 0.21, η^2^= 0.03
Social and physical pleasure	*F*(2,99) = 4.17; *p* = 0.018, η^2^= 0.06	*F*(1,99) = 22.23; *p* = 0.000008, η^2^= 0.17	*F*(2,99) = 1.92; *p* = 0.15, η^2^= 0.03
Arousal/power	*F*(2,99) = 6.77; *p* = 0.002, η^2^= 0.10	*F*(1,99) = 12.65; *p* = 0.0006, η^2^= 0.10	*F*(2,99) = 2.40; *p* = 0.096, η^2^= 0.07
Social assertiveness	*F*(2,99) = 11.8; *p* = 0.000025, η^2^= 0.17	*F*(1,99) = 14.44; *p* = 0.00025, η^2^= 0.10	*F*(2,99) = 1.12; *p* = 0.33, η^2^= 0.02
Relaxation	*F*(2,99) = 2.67; *p* = 0.07, η^2^= 0.05	*F*(1,99) = 13.55; *p* = 0.00040, η^2^= 0.11	*F*(2,99) = 0.87; *p* = 0.42, η^2^= 0.02

### Effect of Age and Alcohol Consumption on IAT Scores

Implicit Association Test results were first analyzed with Student *t*-tests in order to test whether there is an IAT effect for alcohol stimulation and sedation in the whole sample. Significant IAT effects were found for both stimulation, *t*(104) = 9.99, *p* < 0.01, and sedation, *t*(104) = 7.72, *p* < 0.01. Participants were faster to associate stimulation and sedation concepts with alcohol than with soft drinks.

**FIGURE 2:**
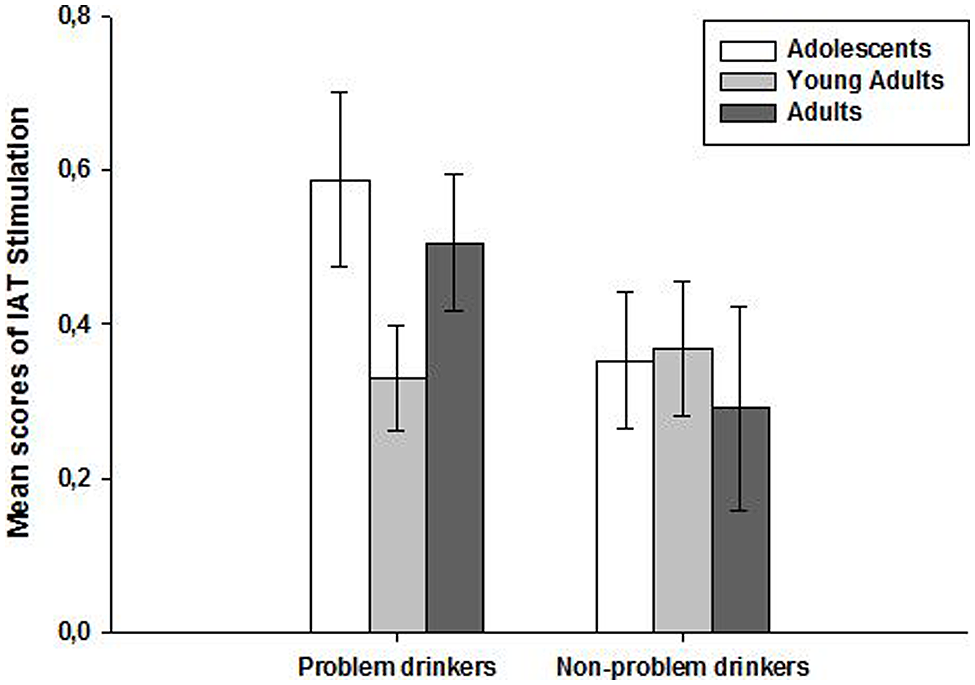
**Mean scores (±SEM) on the Implicit Association Test (IAT) stimulation subscales according to age (adolescents, young adults, and adults) and alcohol consumption groups (problem and non-problem drinkers)**.

**FIGURE 3:**
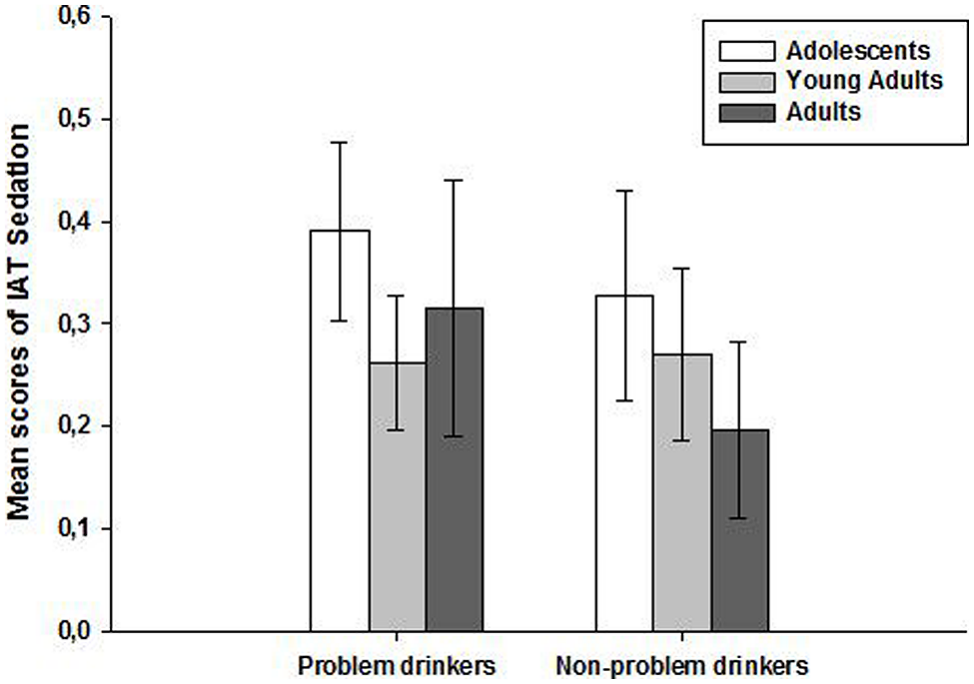
**Mean scores (±SEM) on the IAT sedation subscales according to age (adolescents, young adults, and adults) and alcohol consumption groups (problem and non-problem drinkers)**.

However, the two-way ANOVA computed on the stimulation scores of the IAT (**Figure 2**) failed to show significant main effects of either age [*F*(2,99) = 0.77, *p* = 0.46, η^2^ = 0.02] or the consumption group [*F*(1,99) = 2.95, *p* = 0.09, η^2^ = 0.03]. Moreover, no significant interaction was observed, *F*(2,99) = 1.35, *p* = 0.26, η^2^ = 0.03. Similarly, the two-way ANOVA computed on the sedation scores of the IAT (**Figure 3**) showed no significant main effects of either age [*F*(2,99) = 0.66, *p* = 0.52, η^2^ = 0.01] or consumption group [*F*(1,99) = 0.56, *p* = 0.45, η^2^ = 0.006] and no significant interaction [*F*(2,99) = 0.25, *p* = 0.77, η^2^ = 0.005].

## Discussion

The results of the present study show that positive alcohol explicit expectancies gradually decrease with age. Adolescents aged 13 to 16 report especially high positive alcohol effect expectancies. This effect is statistically significant for all kinds of positive expectancies, with the exception of relaxation expectancies that only show a statistical trend (*p* = 0.07). The results also show that problem drinkers from all age groups report higher positive explicit alcohol expectancies than non-problem drinkers. This effect is similar for all kinds of explicit positive expectancies. A very different pattern of results was obtained with implicit alcohol associations measured with the IAT. While implicit associations between alcohol and stimulation and sedation concepts were found in the whole sample, the present study did not find significant differences between age groups and between problem and non-problem drinkers on those measures.

The results of the present study confirm previous studies showing differences between low and high alcohol drinkers in positive explicit alcohol expectancies (Goldman et al., 1999). However, in contrast to some of those studies, the present results did no show selective differences in some specific positive expectancies. In fact, all positive alcohol explicit expectancies, except relaxation, were significant predictors of alcohol consumption behaviors. As this is a transversal study, the present study cannot solve the question of the causality direction between alcohol positive expectancies and alcohol-related behaviors. One possible explanation is that positive alcohol expectancies exert a causal influence on alcohol drinking, leading to a higher frequency of alcohol consumption and therefore to a higher prevalence of potential alcohol-related problems. Initial positive expectancies about the effects of alcohol may come from family or peer attitudes toward alcohol consumption (Lang and Stritzke, 1993; Zucker et al., 1995; Brown et al., 1999). Previous studies showed that children hold alcohol effect expectancies before they initiate alcohol drinking (Miller et al., 1990; Dunn and Goldman, 1996), confirming that alcohol expectancies are transmitted from other people outside alcohol drinking experiences. The first experiences with alcohol drinking may also contribute to develop various degrees of positive alcohol expectancies. It is well known that people differ in their initial physiological reactions to alcohol and this effect is partially determined by genetic factors (Grant, 1998). Such early acquired positive alcohol expectancies may then influence subsequent alcohol drinking behaviors. However, the causal direction of the relationship between alcohol expectancies and drinking behaviors may be the other way round. It is quite possible that repeated alcohol consumption gradually increases positive expectancies about its effects, eventually leading high drinkers to develop stronger positive expectancies than low drinkers. Such an effect may develop through a self-justification process or as a consequence of the sensitization of the actual positive effects of alcohol consumption. Animal studies clearly showed that some of the positive effects of alcohol, especially its stimulant effects, gradually increase with repeated alcohol administrations (Kayir and Uzbay, 2002; Correa et al., 2003; Didone et al., 2008). This phenomenon is called sensitization and plays a key role is some current addiction theories, such as the incentive-sensitization theory of addiction (Robinson and Berridge, 1993, 2008). It is therefore possible that the stronger positive alcohol expectancies reported by high alcohol drinkers relate to actual “sensitized” positive effects of alcohol they experience when drinking. In the high drinker group of the present study, positive alcohol expectancies are of higher magnitude in the younger participants, which might suggest that such expectancies do not sensitize with further alcohol drinking during aging. However, only longitudinal studies following young participants before they start drinking alcohol will allow testing such hypotheses. Such studies should be able to test whether alcohol expectancies change after the initiation of alcohol consumption and following repeated alcohol consumption episodes, or whether they preexist to the first experience with alcohol.

One important result of the present study is that explicit positive expectancies gradually decrease with age from young adolescence to adulthood. In a previous study on college students aged from 17 to 35, it was shown that subjects under the age of 20 reported more positive expectancies than subjects above the age of 20 (Lundahl et al., 1997). The present study further shows that subjects under the age of 17 report even higher explicit positive alcohol effect expectancies, especially high drinkers. There are several possible explanations to such a decrease in positive alcohol expectancies with age. First, the actual physiological reactions to alcohol may change with age, translating into a more positive experience in young adolescents. To our knowledge, no studies investigated whether the actual positive effects of alcohol consumption decrease with age throughout adolescence in humans. However, results from animal research indicate that alcohol stimulant effects and alcohol reinforcing effects decrease from weaning to adulthood (Stevenson et al., 2008; Quoilin et al., 2010), whereas the sedative effects gradually increase during the same developmental period (Silveri and Spear, 1998; Spear and Varlinskaya, 2005; Quoilin et al., 2010). It is therefore possible that similar effects occur in humans, leading to changes in alcohol effect expectancies. A decrease in alcohol positive effect expectancies with age might also indirectly result from the growing awareness of the adverse effects of alcohol. With the end of adolescence, the pattern of alcohol consumption tends to change with changes in role obligations (family and employment responsibilities; Leonard and Eiden, 2007). Such changes might also indirectly impact on the positive alcohol effect expectancies. Finally, it should be remembered that this is a cross-sectional study. Therefore, we cannot rule out that age differences in positive alcohol effect expectancies result from a cohort effect rather than from developmental changes. Only future longitudinal studies can confirm that positive alcohol effect expectancies evolve with age in the same subjects in a similar way as the behavioral effects of alcohol change with age in animal models (Quoilin et al., 2010).

In contrast to explicit expectancies, implicit alcohol associations were not significantly associated with either alcohol consumption behaviors or age in the present study. As alcohol exerts both stimulant and sedative effects according to the ingested alcohol dose and to the time post-ingestion (Pohorecky, 1977; King et al., 2002), implicit alcohol associations were not tested on the arousal–sedation dimension using a single bipolar IAT task. In contrast, we used two unipolar IAT tasks to separately assess arousal and sedation implicit alcohol associations. This allowed recording the co-existence of arousal and sedation implicit associations in the same participants. For the whole sample, there is a positive and statistically significant correlation (*r* = 0.29; *p* = 0.004) between D statistics for stimulation and sedation alcohol implicit associations. This confirms that participants from the whole sample hold statistically significant implicit alcohol associations for both stimulation and sedation. Such a positive correlation also does not support the idea that sedation and stimulation are two endpoints of a single bipolar dimension of alcohol implicit associations, which would imply a strong negative correlation between both measures. In a study from Wiers et al. (2002), it was shown that heavy and light drinkers differ in their implicit arousal association as measured with the IAT. However, in a previous study from our laboratory, we found no correlation between alcohol implicit arousal association and the levels of current alcohol drinking in a non-selected sample of undergraduate students (Kreusch et al., 2013). A possible explanation to the discrepancies between the results of those studies was that the latter study did not select a sample of heavy alcohol drinkers. However, the present study also did not find significant differences in implicit alcohol stimulation associations between problem and non-problem drinkers from various age groups. Whereas the present study focused on current alcohol consumption, Thush and Wiers (2007) showed that implicit measures predicted future alcohol use over a short period of 1 month. Therefore we cannot rule out that implicit alcohol associations predict future alcohol consumption. However, the present study shows that explicit positive effect expectancies, including arousal expectancies, better predict current levels of alcohol consumption than implicit arousal alcohol associations measured through the IAT. Kreusch et al. (2013) also found that implicit alcohol arousal associations did not correlate with either explicit alcohol arousal expectancies measured through the AEQ or with the subjective stimulant experience reported after alcohol consumption and measured with the biphasic alcohol effects scale (BAES). It was argued that implicit and explicit processes are distinct and differentially influence behavior (Stacy, 1997). According to this view, implicit and explicit measures should predict unique variance in alcohol drinking behaviors. Although explicit expectancies indeed explained a part of the current alcohol drinking variance in the present study, we failed to find a significant contribution of implicit arousal and sedation alcohol associations measured with the IAT in any of the tested age groups.

In summary, the present results show higher positive alcohol explicit expectancies in problem drinkers relative to non-problem drinkers. They also show that such explicit expectancies gradually decrease with age in the three cross-sectional groups of adolescents, young adults, and adults. In contrast implicit alcohol arousal and sedation associations measured with the IAT were not significantly associated with either age or current alcohol drinking behaviors. Explicit alcohol positive expectancies may therefore be used as predictors of current alcohol consumption, especially in young adolescents.

## Conflict of Interest Statement

The authors declare that the research was conducted in the absence of any commercial or financial relationships that could be construed as a potential conflict of interest.
